# Social media use and negative body image perception in Saudi Arabian women

**DOI:** 10.3389/fpubh.2025.1447563

**Published:** 2025-05-21

**Authors:** Maha AlQahtani, Narmeen Shaikh, Khabir Ahmad, Noara Alhusseini

**Affiliations:** ^1^Biostatistics, Epidemiology and Public Health, College of Medicine, Alfaisal University, Riyadh, Saudi Arabia; ^2^Research Department, King Khaled Eye Specialist Hospital, Riyadh, Saudi Arabia

**Keywords:** negative body image, social media addiction, adult females, Saudi Arabia, beauty filters, BMI, digital health

## Abstract

**Background:**

Social media has become deeply embedded in daily life, particularly among females who spend significant time on visual-based platforms such as Snapchat, and Instagram. Exposure to unrealistic beauty standards on these platforms has been linked to negative body image, a growing global public health concern with well-documented psychological and behavioral consequences. While this relationship has been widely studied globally, research within Saudi Arabia, especially among adult women beyond early adulthood, remains limited.

**Objective:**

This study aimed to examine the association between social media use and body image perception among adult females in Saudi Arabia, while accounting for relevant factors such as sociodemographic characteristics, BMI, and beauty filter use.

**Methods:**

An online cross-sectional survey was conducted among adult females (aged ≥ 18) in Saudi Arabia. Validated instruments—the Social Media Addiction Scale-14 and the Body Image Questionnaire—were used to assess social media addiction and negative body image, respectively. Associations were examined using Poisson regression with robust variance.

**Results:**

Of the 1,136 participants, 71.2% were classified as having a negative body image. Participants with social media addiction severity and BMI outside the normal range (underweight, overweight, or obese) had significantly higher rates of negative body image (*p* < 0.001). In contrast, using beauty filters showed no significant association with negative body image.

**Conclusion:**

Negative body image is highly prevalent among adult females in Saudi Arabia and is significantly associated with both social media and higher body mass index. These findings highlight the need for culturally appropriate interventions to improve body acceptance.

## 1 Introduction

Body image (BI) refers to an individual's perception, thoughts, and feelings about their body, encompassing both physical appearance and internalized beliefs regarding shape, size, and attractiveness (1). BI plays a critical role in shaping self-esteem, self-worth, and overall psychological wellbeing. A negative BI is characterized by dissatisfaction with one's appearance and internalized beliefs that link attractiveness to health, success, and social value, particularly among women (2). A growing body of research has connected negative BI to mental health concerns such as depression, anxiety, disordered eating, and reduced physical activity (3–5).

Theoretical frameworks, such as Social Comparison Theory and Objectification Theory, offer important insights into the development of BI concerns. Social Comparison Theory posits that individuals evaluate themselves by comparing their appearance to others, often leading to negative self-evaluations when idealized standards are not met (6). Objectification Theory explains how women internalize societal beauty norms, resulting in self-objectification, body surveillance, and associated psychological distress (7). In the digital age, these dynamics are intensified by SM, which fundamentally reshapes how individuals construct and evaluate their self-image.

Social media (SM) platforms, such as Instagram, Snapchat, Facebook, and TikTok, have revolutionized the way individuals present themselves and interact with others. While these platforms offer opportunities for connection and self-expression, they also expose users to highly curated and digitally enhanced images, particularly those focused on physical appearance. Research consistently demonstrates that frequent exposure to such content is associated with increased self-objectification, internalization of thin ideals, and appearance-based comparisons (8, 9). Visual trends such as “fitspiration” and “thinspiration” reinforce unrealistic beauty standards, contributing to BI dissatisfaction and disordered eating behaviors (10, 11). Moreover, the widespread use of filters and photo-editing tools—especially on visually oriented platforms like Instagram and Snapchat—has amplified these effects. A U.S.-based qualitative study by Eshiet (12) found that filtered images significantly diminished the self-esteem and BI of young women. This growing phenomenon, often referred to as “Snapchat dysmorphia,” reflects how users are increasingly seeking cosmetic procedures to match their digitally altered appearance (13).

Social media addiction (SMA), characterized by excessive, compulsive, and problematic use of social media that interferes with daily functioning, has emerged as a significant public health concern. SMA has been associated with several adverse psychological outcomes, including depression, anxiety, and BI dissatisfaction (14, 15). It also intensifies appearance-related insecurities by increasing exposure to idealized content and reinforcing the internalization of cultural beauty ideals. Activities such as photo editing, posting selfies, and engaging in appearance-based comparisons further exacerbate these effects, particularly when individuals experience a dissonance between their digital identities and their real-life selves (16, 17).

Despite the expanding global literature, research within Middle Eastern contexts, particularly Saudi Arabia, remains limited. Cultural, social, and religious norms may uniquely influence how women experience BI and interact with SM. Some national studies have begun to explore this relationship. For instance, Alanazi et al. (18) found that high Instagram engagement was associated with greater BI dissatisfaction among Saudi females. Similarly, Alruwayshid et al. (19) identified a significant correlation between time spent on SM and BI dissatisfaction. However, these studies are often constrained by narrow age ranges or single-platform analyses, limiting their broader applicability.

Body Mass Index (BMI) is another important variable in BI research, as it influences how individuals perceive their bodies, particularly in comparison to idealized media portrayals. International studies suggest that individuals with higher BMIs are more vulnerable to negative BI when exposed to unrealistic beauty standards (20, 21). In Saudi Arabia, AlSaud et al. (22) reported that women with higher BMIs were more likely to experience negative BI. However, no direct association with SMA was observed, highlighting the complexity of these relationships.

Although previous research has examined the relationship between SM and BI, most existing studies in the Saudi context remain limited in scope. Many have examined only two variables without considering their combined influence. Others have limited their analysis to one or two SM platforms, primarily Instagram or Snapchat, thereby overlooking broader patterns of digital engagement. Additionally, several studies have focused on narrow demographic groups, including adolescents, university students, or solely Saudi nationals (18, 22, 23). These limitations reduce the generalizability of findings and fail to capture the multifaceted nature of BI perception within the diverse female population in Saudi Arabia.

The current study addresses prior gaps by concurrently examining the relationship between SM use, BI perception, and BMI among adult females (18+) in Saudi Arabia, including both Saudi and non-Saudi residents. By considering diverse nationalities, the study emphasizes that sociocultural context, rather than ethnicity, plays a pivotal role in shaping BI. Unlike earlier research limited to specific platforms or age groups, this study captures engagement across all major SM platforms and includes beauty filter usage, enabling a more integrated understanding of digital and individual-level influences. Given Saudi Arabia's high SM penetration—over 82% of the population uses social media daily, with average usage exceeding 3 h (24)—this investigation is both timely and essential for guiding future public health strategies.

The findings of this study have the potential to inform culturally sensitive public health interventions that promote healthy social media use and positive body image. They may also support the design of awareness campaigns and community-based strategies to mitigate SM-related pressures, particularly among women. As social media continues to shape personal and social perceptions of the body, this research offers timely insights for researchers, mental health professionals, and policymakers seeking to address body image concerns in the digital era.

### 1.1 Research question

What is the association between social media use and body image perception among adult females in Saudi Arabia?

### 1.2 Objectives

- Assessing the prevalence of negative body image among adult females residing in Saudi Arabia.
- Examining the associations between negative body image and selected factors, including sociodemographic characteristics, social media addiction, beauty filter use, BMI, and number of children.

## 2 Methods

### 2.1 Study design and setting

This study employed a cross-sectional design targeting adult females aged 18 years and older residing in Saudi Arabia, regardless of nationality. Data were collected between November 2022 and January 2023 using a bilingual (Arabic and English) questionnaire distributed via Google Forms. The study focused on adult females living in Saudi Arabia, including both Saudi nationals and non-Saudi residents. Accordingly, the survey was administered in both Arabic and English to reflect the population's bilingual nature and ensure clarity, inclusivity, and cultural appropriateness. This bilingual approach was intentional and integral to the study design, aiming to enhance accessibility and ensure accurate responses from participants across linguistic backgrounds. The survey link was shared through various social media platforms, including WhatsApp, LinkedIn, X (formerly Twitter), and Telegram. These platforms were selected based on their accessibility, practicality, and potential to reach a broader, more responsive adult audience.

### 2.2 Participants and sample size

Participants were recruited using non-probability voluntary response sampling. The required sample size was calculated using OpenEpi (25). Based on an estimated population of 11 million adult females in Saudi Arabia (26), a 73% prevalence of negative body image (22), a 95% confidence level, and a 5% absolute precision, the minimum sample size required was 385 participants. However, the final analysis included 1,136 participants who met the eligibility criteria to enhance the study's statistical power and representativeness.

Inclusion criteria were adult females (≥18 years) residing in Saudi Arabia. Pregnant women were excluded due to the potential impact of pregnancy-related physical and psychological changes on body image perception (27).

### 2.3 Data collection tools

The questionnaire was bilingual (Arabic and English) and was distributed via Google Forms, as outlined in Section 2.1.

The survey comprised three main sections: (1) Sociodemographic Data: Age, education, occupation, monthly income, marital status, and self-reported weight/height (for BMI). (2) Social Media Addiction Scale: The 14-item SMAS. (3) Body Image Questionnaire: The nine-item BIQ.

Additionally, participants were asked about the average daily time spent on social networking sites (SNSs), the frequency of use of specific apps, the perceived negative influence of these apps on body image, the frequency of using beauty filters, and overall body image satisfaction.

### 2.4 Validity and reliability

Validated instruments were used to assess both social media addiction (SMA) and body image (BI). The 14-item Social Media Addiction Scale (SMAS-14) has been previously validated in the Arab context (28). The original questionnaire was developed in English and translated into Arabic using forward and backward translation by bilingual experts, following standard procedures to ensure linguistic and conceptual equivalence. To assess clarity and cultural relevance, a pilot study was conducted with 20 participants from diverse linguistic backgrounds. Based on their feedback, minor adjustments were made, and these participants were subsequently excluded from the main analysis. Internal consistency was evaluated using Cronbach's alpha, calculated separately for each language version. The SMAS-14 yielded a coefficient of 0.84 and 0.85 for Arabic and English versions, respectively.

The BIQ-9, a 9-item self-report tool developed by Maynard (29) and published on HealthyPlace, assesses body image concerns. Though not peer-reviewed, it has been used in research, such as AlSaud et al. (22) with Saudi females. We used the unmodified English version and developed an Arabic version through a forward-backward translation process by bilingual experts to ensure linguistic accuracy and conceptual equivalence. Both versions were piloted with 20 adult females to confirm clarity, readability, and cultural relevance. In our study, the English version showed strong internal consistency (Cronbach's alpha = 0.82), and the Arabic version demonstrated good internal consistency (Cronbach's alpha = 0.80).

### 2.5 Variables

#### 2.5.1 Dependent variables

Body Image (BI) was assessed using the validated 9-item “yes/no” Body Image Questionnaire (BIQ), developed by Maynard (29). The questionnaire comprises nine questions, each requiring a “yes” (scored as 1) or “no” (scored as 0) response, with scores ranging from 0 to 9. A score of ≥3 on the BIQ identified participants with a negative BI.

#### 2.5.2 Independent variables

- Demographics: age, education level, employment status, monthly income, marital status, and number of children.
- Body Mass Index (BMI): Calculated from self-reported weight (kg) and height (m).
- Social Media Addiction (SMA) was measured using the 14-item Social Media Addiction Scale (SMAS) developed by Al-Menayes (28), a modified version of the Internet Addiction Test (IAT) created by Young in 2010. Participants responded on a 5-point Likert scale: strongly agree = 5, agree = 4, neutral = 3, disagree = 2, strongly disagree = 1, with the fifth item reverse-scored. Scores on the SMA severity scale, which ranges from 0 to 70, were classified according to previously established addiction score categories (28), utilizing proportional cutoffs consistent with the original categorization. Participants scoring 0–22 were categorized as having no SMA, those scoring 23–34 as mildly addicted, those scoring 35–55 as moderately addicted, and those scoring 56–70 as severely addicted.

### 2.6 Data analysis

The data were analyzed using STATA 18.0 [StataCorp LLC, College Station, TX, USA]. BMI categories were defined according to the World Health Organization (WHO) criteria: underweight ( ≤ 18.49), normal weight (18.50–24.99), overweight (25.00–29.99), and obese (≥30.00). Categorical data were reported as frequencies and percentages.

Negative body image was treated as a binary outcome (score ≥3 vs. score < 3). Factors associated with negative body image were examined using Poisson regression with robust variance. Prevalence ratios (PR) and 95% confidence intervals (CIs) were reported for both unadjusted and adjusted models. This method is a better alternative to logistic regression for analyzing binary outcomes in cross-sectional studies because it provides accurate estimates, and the prevalence ratio is more interpretable and easier to communicate to non-specialists than the odds ratio (31). Covariates with a *p*-value ≤ 0.2 (indicating that at least one category was ≤ 0.2 for multiple category variables) in the bivariate analysis were included in the multivariable analysis. A *p*-value of < 0.05 was considered statistically significant.

### 2.7 Ethical consideration

Ethics approval was obtained from the Institutional Review Board (IRB) of Alfaisal University (IRB approval number: 20179) on November 8, 2022. Participation in the study was voluntary, and participants could withdraw at any time. Data were anonymized to protect privacy; no identifying personal information, such as names or emails, was collected. Completing the questionnaire was considered informed consent.

## 3 Results

### 3.1 Socio-demographic and socio-economic characteristics of survey participants

A total of 1,136 females completed the online survey (Table 1). Most participants (*n* = 641, 56.4%) were aged 18 to 29, while another 298 (26.2%) were aged 30 to 39. Most participants (*n* = 847, 74.6%) had bachelor's degrees, whereas 123 (10.8%) had completed higher education. Over one-third of the respondents (40.1%) were employed, while 26.8% were unemployed; 29.9% had a monthly income of less than SAR 5,000, and 40.4% reported no income. Additionally, 39.7% (451 participants) were married, and 38.7% (440 respondents) had children. Almost half of the participants (45.7%) reported a normal BMI, while 25.3% and 20.2% were classified as overweight and obese, respectively.

**Table 1:** Socio-demographic characteristics of the study participants (*n* = 1,136).

**Characteristic**		**Count**	**%**
Age, year	18–29	641	56.4
	30–39	298	26.2
	40–49	136	12.0
	≥50	61	5.4
Education	High school or less	166	14.6
	College degree	847	74.6
	Higher education	123	10.8
Employment status	Student	352	31.0
	Employed	456	40.1
	Unemployed	305	26.8
	Retired	23	2.0
Monthly income, SAR	No income	459	40.4
	< 5,000	340	29.9
	5,000 to ≤ 10,000	152	13.4
	>10,000 to ≤ 20,000	113	9.9
	>20,000	19	1.7
	I do not want to disclose	53	4.7
Current marital status	Single	621	54.7
	Married	451	39.7
	Divorced	57	5.0
	Widow	7	0.6
Having children	No	696	61.3
	Yes	440	38.7

### 3.2 Social networking site usage and satisfaction with body image

Table 2 illustrates participants' daily usage of social networking sites (SNSs). The vast majority (72.5%) reported spending four or more hours per day on SNSs, while 24.1% reported 1–3 h, and only 3.5% reported less than an hour. The most commonly used SNSs were WhatsApp (67.9%), Snapchat (66.3%), TikTok (49.0%), Instagram (41.2%), and X (35.2%). When asked which apps negatively affected their body image (BI), 36.4% identified Snapchat, 30.2% TikTok, and 26.3% Instagram.

**Table 2:** Time spent on SNS daily, most frequently used SNS, and other characteristics of users.

**Characteristic**		**Count**	**%**
Time spent on SNS daily, hours	< 1	39	3.5
	1–3	272	24.1
	4–6	402	35.6
	7–9	220	19.5
	>9	197	17.4
The most frequently used SNS	WhatsApp	767	67.9
	Snapchat	749	66.3
	TikTok	554	49.0
	Instagram	466	41.2
	X platform	398	35.2
	YouTube	28	2.5
	Facebook	18	1.6
	Other	46	4.1
The app that negatively affects your body perception	Snapchat	414	36.4
	TikTok	343	30.2
	Instagram	299	26.3
	X platform	59	5.2
	Facebook	12	1.1
	YouTube	4	0.4
	Other	6	0.5
How often do you apply social media app filters?	Never	165	14.6
	Almost never	133	11.8
	Occasionally/Sometimes	354	31.3
	Almost every time	230	20.4
	Every time	248	21.9
Overall, body image satisfaction levels	Extremely dissatisfied	95	8.4
	Slightly dissatisfied	167	14.7
	Neutral	169	14.9
	Slightly satisfied	409	36.0
	Extremely satisfied	296	26.1

Regarding the use of social media filters, 31.3% of participants reported using them “sometimes,” 20.4% “almost every time,” and 21.9% “every time.” In contrast, 26.4% reported “rarely” or “never” using filters. As for body image satisfaction, 26.1% of participants were extremely satisfied, while 36.0% were slightly satisfied. The remaining participants were either neutral (14.9%), slightly dissatisfied (14.7%), or highly dissatisfied (8.4%).

### 3.3 Social media addiction

**Figure 1:**
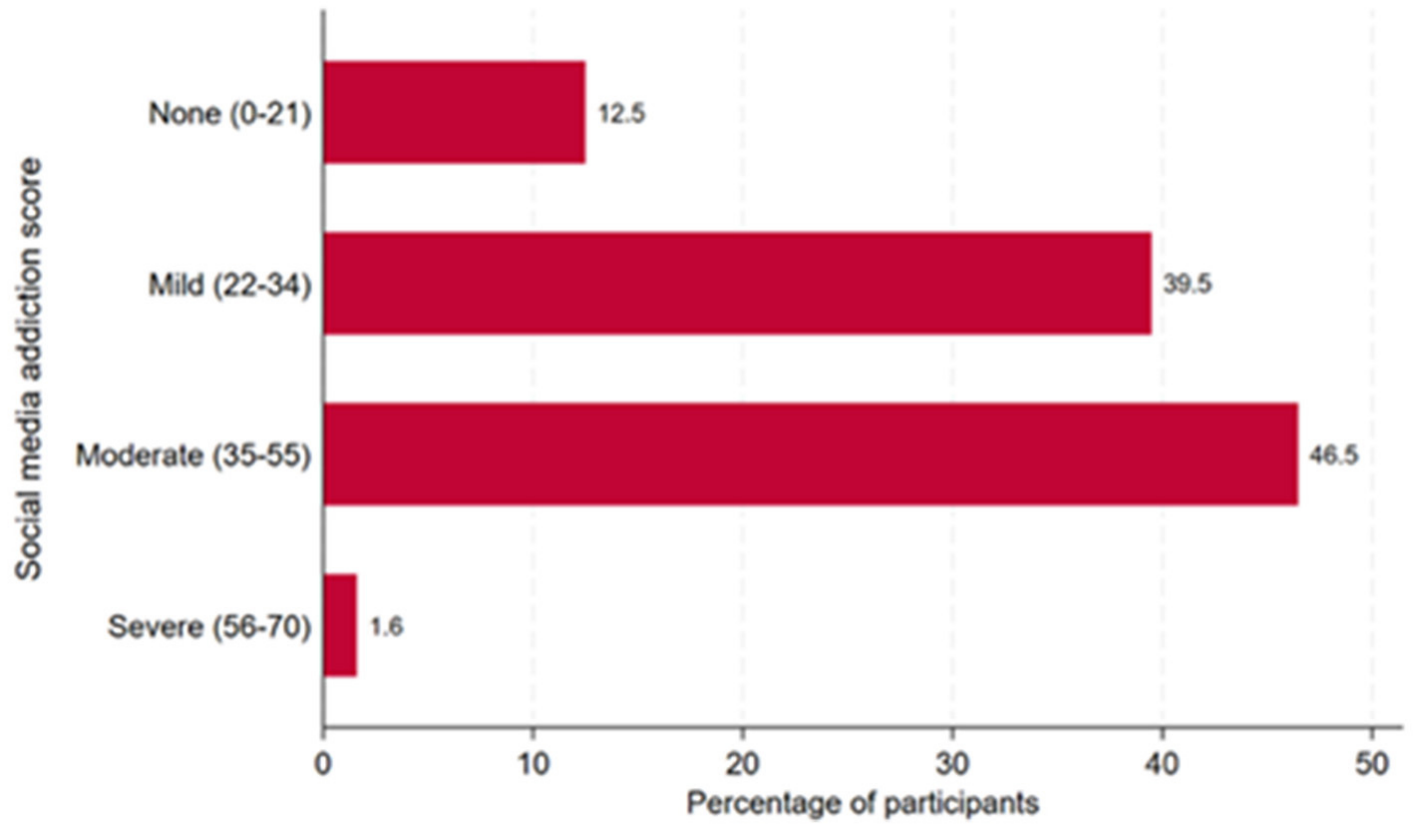
Social media addiction categories among survey participants (*n* = 1,136).

Figure 1 presents the distribution of social media addiction (SMA) severity among participants (*n* = 1,130). A total of 12.5% exhibited no signs of SMA, 39.5% were classified as mildly addicted, 46.5% as moderately addicted, and 1.6% as severely addicted.

### 3.4 Prevalence of negative body image, overall and by subgroup

**Figure 2:**
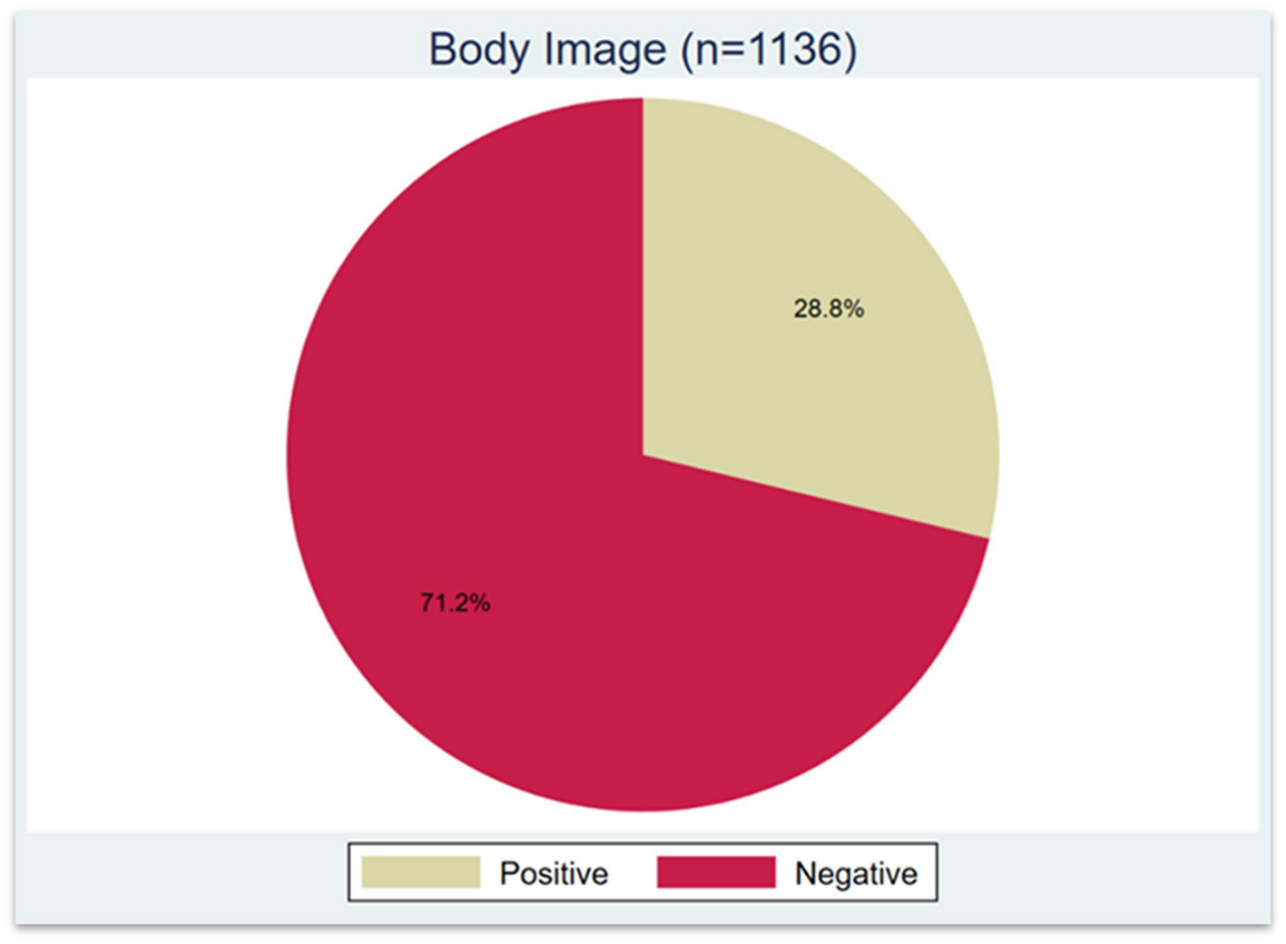
Prevalence of negative BI among survey participants (*n* = 1,136).

More than two-thirds of participants (71.2%) had negative perceptions of their BI (Figure 2). According to Table 3, the prevalence of negative BI did not significantly differ by age, education level, occupation, income, or marital status. However, it increased notably with SMA severity: 69.1% among those with mild SMA, 78.3% with moderate SMA, and 94.4% with severe SMA. The prevalence of negative BI was also higher among participants who reported more frequent use of SM filters, rising from 63.2% among almost-never users to 81.9% among those who used filters every time. Similarly, as BMI increased, so did the prevalence of negative BI, from 62.8% in those with normal weight to 90.4% in obese participants.

**Table 3:** Prevalence of negative BI among survey participants (*n* = 1,136).

**Characteristic**		**Number of participants**	**Individuals with negative BI**	
			**Freq**.	**%**
All		1,136	809	71.2
Age, year	18–29	641	456	71.1
	30–39	298	215	72.1
	40–49	136	91	66.9
	≥50	61	47	77.0
Education	High school or less	166	121	72.9
	College degree	847	597	70.5
	Higher education	123	91	74.0
Employment status	Employed	456	327	71.7
	Unemployed	305	219	71.8
	Retired	23	15	65.2
	Student	352	248	70.5
Monthly income, SAR	No income	459	326	71.0
	< 5,000	340	252	74.1
	5,000 to ≤ 10,000	152	108	71.1
	>10,000 to ≤ 20,000	113	80	70.8
	>20,000	19	12	63.2
	I do not want to disclose	53	31	58.5
Marital status	Single	621	431	69.4
	Married	451	329	72.9
	Divorced	57	44	77.2
	Widow	7	5	71.4
SMA	None (Score < 22)	141	69	48.9
	Mild (Score 22–34)	446	308	69.1
	Moderate (Score 35–55)	525	411	78.3
	Severe (Score 56–70)	18	17	94.4
Frequency of social media app filter use	Never	165	113	68.5
	Almost never	133	84	63.2
	Occasionally/Sometimes	354	233	65.8
	Almost every time	230	172	74.8
	Every time	248	203	81.9
BMI	Normal	519	326	62.8
	Underweight	101	43	42.6
	Overweight	287	233	81.2
	Obese	229	207	90.4

### 3.5 Poisson regression analysis of factors associated with negative BI among participants

Out of the nine covariates analyzed in the bivariate Poisson regression for their association with negative BI, only four met the threshold for statistical significance and were included in the multivariable analysis: SMA severity, frequency of social media beauty filter use, BMI, and number of children (Table 4).

**Table 4:** Poisson regression analysis of factors associated with negative BI among participants.

**Characteristic**		**Univariate analysis**		**Multivariable analysis**	
		**Prevalence ratio (95% CI)**	***P***	**Prevalence ratio (95% CI)**	***P***
Age, year	18–29	1.00			
	30–39	1.01(0.93,1.11)	0.749		
	40–49	0.94(0.83,1.07)	0.349		
	≥50	1.08 (0.94,1.25)	0.283		
Education	High school or less	1.00			
	College degree	0.97 (0.87,1.07)	0.521		
	Higher education	1.01 (0.88, 1.17)	0.835		
Employment status	Employed	1.00			
	Unemployed	1.00 (0.91, 1.10)	0.978		
	Retired	0.91 (0.67,1.23)	0.541		
	Student	0.98(0.90,1.07)	0.697		
Monthly income, SAR	No income	1.00			
	< 5,000	1.04 (0.96, 1.14)	0.330		
	5,000 to ≤ 10,000	1.00 (0.89, 1.12)	0.995		
	>10,000 to ≤ 20,000	1.00 (0.87,1.14)	0.962		
	>20,000	0.89 (0.63, 1.26)	0.509		
	I do not want to disclose	0.82 (0.65, 1.04)	0.104		
Current marital status	Single	1.00			
	Married	1.05 (0.97, 1.13)	0.203		
	Divorced	1.11 (0.96, 1.29)	0.166		
	Widow	1.03 (0.64, 1.65)	0.905		
SMA	None (Score < 22)	1.00		1.00	
	Mild (Score 22–34)	1.41 (1.18, 1.69)	< 0.001	1.45 (1.22, 1.72)	**< 0.001**
	Moderate (Score 35–55)	1.60 (1.34, 1.90)	< 0.001	1.61 (1.36, 1.91)	**< 0.001**
	Severe (Score 56–70)	1.93 (1.58, 2.36)	< 0.001	1.74 (1.41, 2.14)	**< 0.001**
Frequency of beauty filter use^*^	Never	1.00		1.00	
	Almost never	0.92 (0.78, 1.09)	0.339	0.90 (0.77, 1.05)	0.165
	Occasionally/Sometimes	0.96 (0.85, 1.09)	0.543	0.94 (0.83, 1.06)	0.332
	Almost every time	1.09 (0.96, 1.24)	0.178	1.02 (0.90, 1.15)	0.796
	Every time	1.20 (1.06, 1.35)	0.003	1.10 (0.98, 1.24)	0.111
BMI	Normal	1.00		1.00	
	Underweight	0.68 (0.54, 0.86)	0.001	0.65 (0.52, 0.82)	**< 0.001**
	Overweight	1.29 (1.19, 1.41)	< 0.001	1.31 (1.20, 1.42)	**< 0.001**
	Obese	1.44 (1.33, 1.56)	< 0.001	1.48 (1.37, 1.61)	**< 0.001**
Number of children	No children	1.00		1.00	
	1–2	1.01 (0.90, 1.12)	0.922	0.92 (0.83–1.03)	0.144
	3–5	1.01(0.88, 1.15)	0.936	0.91 (0.83–1.00)	**0.040**
	≥6	1.15 (0.97, 1.37)	0.098	1.06 (0.90–1.24)	0.481

^*^Beauty filters on social media are also known as appearance-altering filters.

^*^Bold p-values indicate statistically significant results.

In the multivariable analysis, SMA severity, BMI, and number of children were significantly associated with negative BI, while the frequency of social media beauty filter use was not.

Women with mild, moderate, and severe SMA were 1.45 times (adjusted PR, 1.45, 95% CI, 1.22–1.72; *p* < 0.001), 1.61 times (adjusted PR, 1.61, 95% CI, 1.36–1.91; *p* < 0.001), and 1.74 times (adjusted PR, 1.74, 95% CI, 1.41–2.14; *p* < 0.001) more likely, respectively, to have negative BI compared to women with no SMA, while adjusting for the effects of beauty filter use, BMI, and number of children.

Compared to women with a normal BMI, those who were underweight had a significantly lower prevalence of negative BI (adjusted PR, 0.65, 95% CI, 0.52–0.82; *p* < 0.001), while those who were overweight or obese had a significantly higher prevalence (adjusted PR, 1.31, 95% CI, 1.20–1.42; *p* < 0.001, and adjusted PR, 1.48, 95% CI, 1.37–1.61; *p* < 0.001, respectively), adjusting for SMA severity, beauty filter use, and number of children.

Furthermore, women with 3–5 children were less likely to report negative BI (adjusted PR, 0.91, 95% CI, 0.83–1.00; *p* = 0.040) compared to those with no children, whereas the associations for 1–2 children (*p* = 0.144) and ≥ 6 children (*p* = 0.481) were not statistically significant.

## 4 Discussion

This study investigated the prevalence of negative body image (BI) and social media addiction (SMA) among adult females in Saudi Arabia. It also examined their associations with BMI, beauty filter usage, and the number of children. The findings reveal distinct patterns in SMA and BI concerns, highlighting the complex role of digital engagement in shaping women's body perceptions. Each of these factors is discussed below in relation to the study's findings and existing literature.

Consistent with previous national research (22), our findings show that over two-thirds of participants experienced negative BI, reinforcing the growing concerns related to body perception among Saudi women. Notably, while only 23.1% of participants self-reported being slightly or extremely dissatisfied with their body image, a more comprehensive assessment using the validated Body Image Questionnaire (BIQ) revealed that 71.2% met the criteria for negative BI. This substantial gap suggests that brief self-assessment items may underestimate the true prevalence of negative BI. It also highlights the potential role of social desirability bias, whereby individuals may underreport dissatisfaction to align with perceived cultural or societal expectations. These findings reinforce the importance of using validated instruments to capture nuanced perceptions of BI in research.

In our study, SMA was prevalent in varying degrees among respondents, with the majority falling into the “mild” and “moderate” categories, and only a minority (1.6%) reporting severe SMA. These proportions closely mirror those found in earlier studies conducted in Riyadh, indicating consistency in SMA patterns among Saudi females (22).

Multivariable Poisson regression analysis identified a significant association between SMA and negative BI, corroborating findings from international contexts such as Australia (8) and Malaysia (32). The discrepancy with AlSaud et al. (22), who found no significant link, may stem from their study population being predominantly medical students who reported using social media for academic rather than aesthetic or entertainment purposes.

Interestingly, over one-third of participants identified Snapchat as having a negative impact on their body image. Despite this perception, Snapchat remained one of the most frequently used platforms, second only to WhatsApp. This pattern aligns with previous international research suggesting that highly visual platforms like Snapchat may contribute to negative psychological outcomes due to features like beauty filters (33). These filters, which enable users to manipulate their appearance, may amplify the gap between the actual and ideal self, as posited by Self-Discrepancy Theory (34). This mechanism has been supported in a recent Saudi-based study, which found that while Snapchat filters may temporarily enhance confidence, they can simultaneously generate self-discrepancy and contribute to negative BI (35).

Although some previous studies found an association between beauty filter use and negative BI, our analysis did not reveal a statistically significant link. This contrasts with both local and international research, such as Alsaggaf (35) and Eshiet (12), which reported that frequent use of beauty filters was linked to lower self-esteem and negative body image. A possible explanation for this discrepancy may lie in differences in study populations or filter usage patterns. These findings underscore the need for further research to disentangle the contextual and cultural factors that may shape how beauty filters impact BI across different populations.

Approximately 45.7% of participants were found to have a normal BMI, while 25.3% were overweight and 20.2% were classified as obese. The obesity rate aligns closely with national estimates reported by the Saudi Ministry of Health 2019 report (30) which indicated that 21% of adult Saudi females were obese. However, our sample's proportion of overweight individuals was somewhat lower than the national average of 33%, possibly due to sampling differences or demographic variation.

Multivariable Poisson regression analysis revealed a significant association between BMI and negative BI. Compared to participants with a normal BMI, those who were underweight, overweight, or obese were all significantly more likely to report negative BI. While previous studies have primarily examined the association between higher BMI and aspects of body dissatisfaction or negative body image (22, 36, 37), our findings offer additional insight by showing that deviations in either direction from a normal BMI are significantly associated with negative BI. This underscores the need for public health initiatives that address BI concerns across all BMI categories, not just among individuals with overweight or obesity.

This study's association between the number of children and negative BI revealed a distinctive pattern. Women with three to five children were significantly more likely to report negative BI compared to those with no children, whereas no significant associations were observed among women with one to two children or those with six or more. This finding may reflect the cumulative physical and psychosocial demands associated with mid-sized families, such as weight retention, limited time for self-care, and increased responsibilities, that may become more pronounced during this transitional phase. In contrast, women with fewer children may retain greater autonomy over their routines, while those with larger families may have adapted to bodily changes or developed stronger coping mechanisms. While direct research on this specific pattern is limited, these findings underscore the importance of addressing BI concerns in relation to SM use and the broader context of women's life stages and family roles.

### 4.1 Limitations and strengths

This study has several limitations. First, its cross-sectional design precludes causal inference; whether negative BI preceded or resulted from SMA, BMI changes, or beauty filter use remains unclear. Second, generalizability may be limited, as the sample included only adult women who actively use SM, excluding non-users. Third, reliance on self-reported data may introduce recall bias, particularly in reporting weight, height, and screen time. Additionally, online self-administered surveys are susceptible to variability in respondents' attention and motivation, potentially affecting data accuracy. Finally, social desirability bias may have influenced responses; for instance, while 62.1% reported satisfaction with their body image, 71.2% were classified as having a negative body image, suggesting a potential gap between self-perception and socially acceptable reporting.

Nonetheless, the study offers several strengths. It is the first in Saudi Arabia to explore the association between SM use and BI among adult females while accounting for BMI, beauty filter use, and number of children. The large sample size (*N* = 1,136) enhances statistical power, and the use of validated instruments to assess both SMA and BI strengthens the reliability of the findings.

## 5 Conclusion

Social media use continues to shape how women perceive and evaluate their bodies, with increasing evidence pointing to its psychological impact. In this study, 71.2% of adult females in Saudi Arabia were classified as having negative BI, a striking prevalence that underscores the urgency of addressing this public health concern. Our findings demonstrate that higher levels of SMA and BMI outside the normal range are significantly associated with negative BI.

Although many participants perceived Snapchat as negatively influencing their body perception, no statistically significant association was found between filter use and BI. This suggests that broader comparison patterns and the internalization of unrealistic ideals may play a more critical role than filter use alone.

These findings highlight the need to integrate BI awareness into both clinical practice and public health initiatives. Healthcare and mental health professionals may benefit from implementing BI screening tools, particularly for women with high SMA or non-normative BMI. Promoting healthier digital behaviors, fostering realistic beauty standards, and encouraging self-acceptance should be prioritized through culturally tailored education and outreach.

Given social media's pervasiveness and influence on self-image, this research offers actionable insights for policymakers, educators, healthcare professionals, and social media platform developers. Designing and implementing targeted interventions, such as awareness campaigns, digital wellness tools, and educational initiatives, can help create a more body-positive environment and support women's psychological wellbeing in the digital age.

## Data Availability

The raw data supporting the conclusions of this article will be made available by the authors, without undue reservation.
